# Early Repolarization Syndrome; Mechanistic Theories and Clinical Correlates

**DOI:** 10.3389/fphys.2016.00266

**Published:** 2016-06-30

**Authors:** Ben N. Mercer, Gordon A. Begg, Stephen P. Page, Christopher P. Bennett, Muzahir H. Tayebjee, Saagar Mahida

**Affiliations:** ^1^West Yorkshire Arrhythmia Service, Leeds General InfirmaryLeeds, UK; ^2^Regional Inherited Cardiovascular Conditions Service, Leeds General InfirmaryLeeds, UK

**Keywords:** early repolarization, ventricular fibrillation, sudden cardiac death

## Abstract

The early repolarization (ER) pattern on the 12-lead electrocardiogram is characterized by J point elevation in the inferior and/or lateral leads. The ER pattern is associated with an increased risk of ventricular arrhythmias and sudden cardiac death (SCD). Based on studies in animal models and genetic studies, it has been proposed that J point elevation in ER is a manifestation of augmented dispersion of repolarization which creates a substrate for ventricular arrhythmia. A competing theory regarding early repolarization syndrome (ERS) proposes that the syndrome arises as a consequence of abnormal depolarization. In recent years, multiple clinical studies have described the characteristics of ER patients with VF in more detail. The majority of these studies have provided evidence to support basic science observations. However, not all clinical observations correlate with basic science findings. This review will provide an overview of basic science and genetic research in ER and correlate basic science evidence with the clinical phenotype.

## Introduction

**Figure 1:**
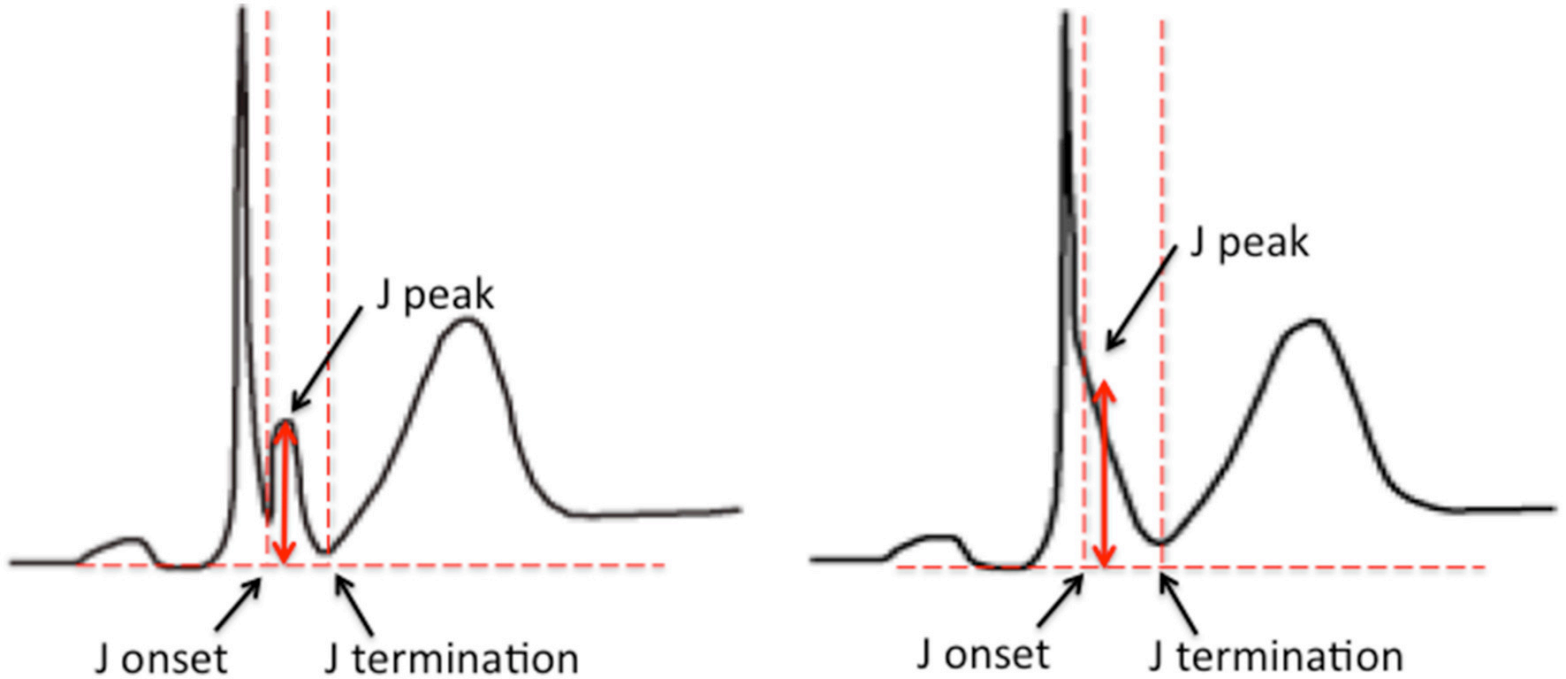
**ER pattern on the surface ECG**. The examples illustrate an end-QRS notch **(left)** and slur **(right)** on the downslope of a prominent R-wave. Current criteria for a diagnosis of ER include; (1) end-QRS notch or slur on the downslope of a prominent R-wave, (2) J point is ≥0.1 mV in 2 or more contiguous leads, excluding leads V_1_–V_3_, and (3) QRS duration is < 120 ms (Figure 1; MacFarlane et al., 2015).

The early repolarization pattern (ER) on the 12-lead electrocardiogram (ECG) is characterized by elevation of the J point in the inferior and/or lateral leads. The J point refers to the junction between the end of the QRS complex and the start of the ST segment. Elevation of the J point typically manifests as a slurring or notching of the terminal part of the QRS complex. Over the years the description of the ER pattern has varied significantly. Based on the most recent expert consensus, in order for ER to be present, the following criteria have to be met; (1) end-QRS notch or slur on the downslope of a prominent R-wave, (2) J point is ≥0.1 mV in 2 or more contiguous leads, excluding leads V_1_–V_3_, and (3) QRS duration is < 120 ms (MacFarlane et al., 2015; Figure 1).

The ER pattern is frequently observed in the general population, with a reported prevalence of 2–31% (Merchant et al., 2009; Maury and Rollin, 2013) but is probably closer to 5–10%. For decades, ER was regarded as a benign ECG variant in healthy populations (Goldman, 1953; Wasserburger and Alt, 1961). However, in recent years accumulating evidence has challenged this view. Multiple case-control and population-based studies have demonstrated that ER is associated with an increased risk of ventricular fibrillation (VF) and sudden cardiac death (SCD; Otto et al., 1984; Garg et al., 1998; Kalla et al., 2000; Horigome et al., 2003; Takeuchi et al., 2003; Haïssaguerre et al., 2008; Sinner et al., 2010; Derval et al., 2011; Haruta et al., 2011; Olson et al., 2011; Rollin et al., 2012; Rosso et al., 2012; Aizawa et al., 2013; Wu et al., 2013). It is important to emphasize however, that ER is common in the general population, and only a small subset of patients with the ECG pattern have an increased risk of SCD. The reported association between ER pattern and VF in these studies has led to the acknowledgment of a clinical syndrome, labeled the early repolarization syndrome (ERS).

The emergence of reports linking ER and SCD has led to significant interest into the pathophysiological basis of the ECG pattern. An in-depth understanding of the mechanistic link between ER and ventricular arrhythmogenesis is likely to have important implications for risk stratification and potentially therapy. This review will provide an overview of mechanistic and genetic research in ERS and correlate basic scientific observations with the clinical phenotype.

## Basic studies in animal models and the repolarization theory

**Figure 2:**
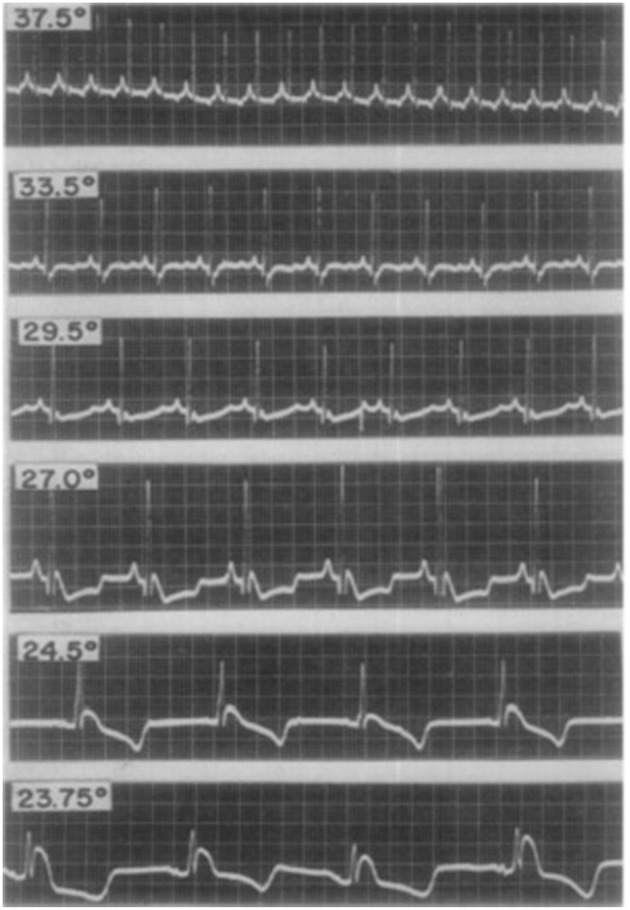
**Electrocardiographic changes from early studies in a dog with experimental hypothermia**. Progressively lower temperatures (centigrade) were observed to be associated with more pronounced J point elevation with a notch in the terminal portion of the QRS complex. Reproduced with permission (Santos and Kittle, 1958).

The first studies exploring the mechanistic basis of J point elevation on the ECG emerged in the 1950's (Osborn, 1953). Osborn reported the appearance of J point elevation secondary to severe hypothermia and acidosis in a canine model. Although it was unclear which of the two factors caused the ECG change, he speculated that the appearance of J waves (in lead II of the surface ECG) represented a “current of injury.” J point elevation was consistently associated with VF in the hypothermic dogs. Santos et al subsequently reported inferior J point elevation (also in lead II) in hypothermic dogs; see Figure 2. However, J point elevation was not associated with acidosis and the authors concluded that the pattern was induced by hypothermia (Santos and Kittle, 1958).

In order to correlate the appearance of J waves on the ECG with the ventricular action potential (AP), West *et al* performed a series of experiments in anesthetized open-chest dogs. They demonstrated that induced hypothermia resulted in J point elevation in ECG lead II with a progressively more pronounced phase 1 “notch” of the ventricular AP. The magnitude of J point elevation was directly proportional with the magnitude of the notch. Furthermore, the AP notch and J point elevation were rate dependent, with more pronounced changes at slower heart rates (West et al., 1959). Based on the observed response of the notch to changing heart rates, the authors argued that J point elevation was unlikely to represent a “current of injury.”

A more in-depth insight into the mechanistic basis of J point elevation came almost four decades later from an elegant series of experiments from Antzelevitch et al. (Yan and Antzelevitch, 1996). In an *ex vivo* model of arterially perfused canine left and right ventricular wedges, they simultaneously recorded transmural ECGs and endocardial, epicardial, and mid-myocardial APs and correlated J waves with transmural AP characteristics (Yan and Antzelevitch, 1996). Consistent with the observations from West et al. hypothermia simultaneously resulted in an increase in the J wave amplitude and a more prominent phase 1 notch in the epicardial AP but not the endocardial AP. They also demonstrated that direct pharmacological inhibition of the I_to_ current with 4-AP resulted in a reduction in the epicardial AP notch and corresponding reduction in the magnitude of the J wave. Furthermore, pacing the ventricular wedges with progressively more premature stimuli resulted in a progressive reduction in the amplitude of the J wave as well as the epicardial phase 1 AP notch. The observation that shortening the pacing cycle length attenuated the J wave in the transmural ECG was attributed to a progressively slower recovery of the I_to_ current at shorter cycle lengths. These observations suggest that a J wave is a manifestation of a transmural voltage gradient created by a more prominent epicardial AP notch relative to the endocardial AP notch. Furthermore, the I_to_ current is an important mediator of the transmural heterogeneity of the phase 1 notch. Overall the findings from these experiments form the basis of the repolarization theory for ERS.

**Figure 3:**
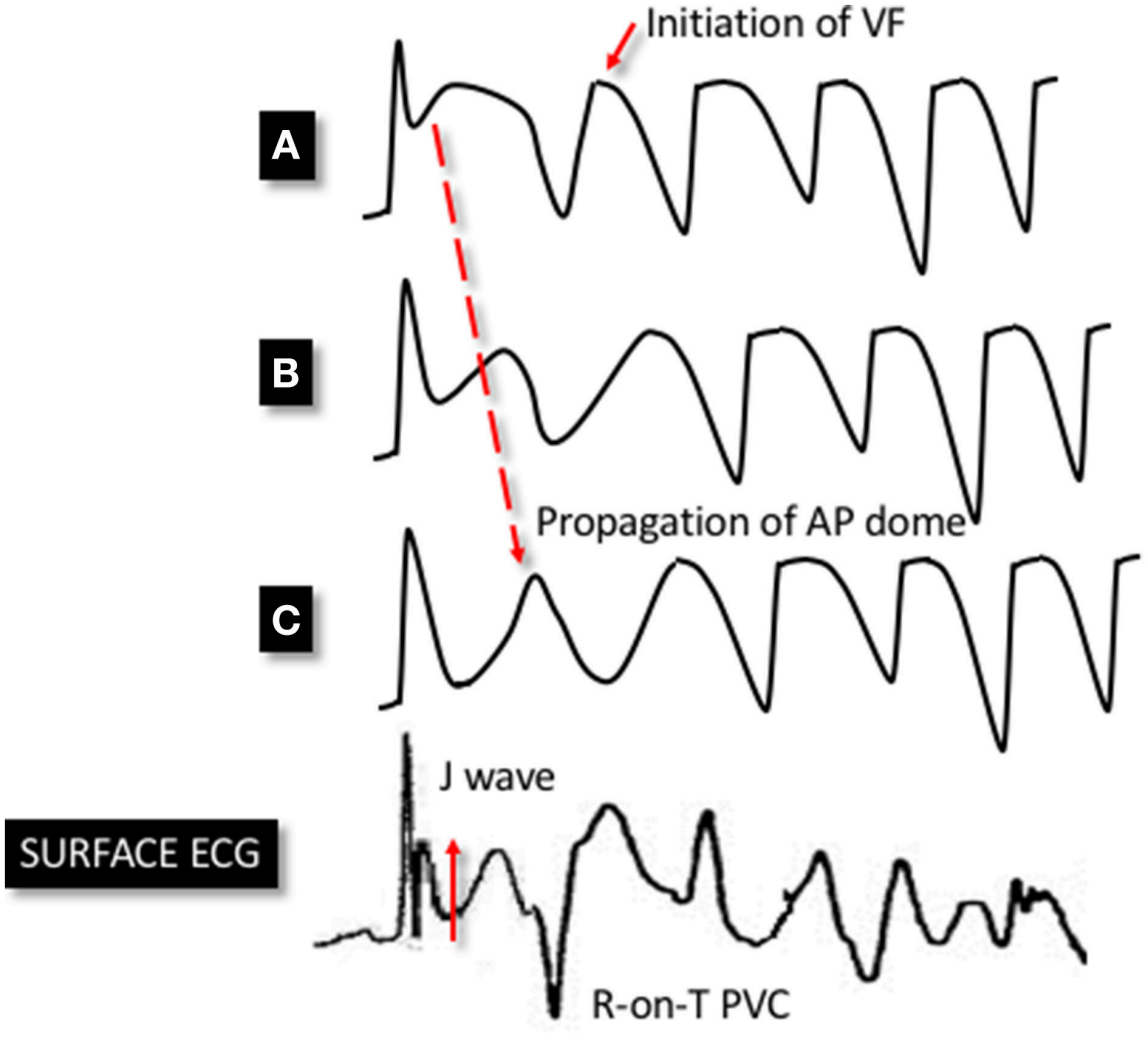
**Schema demonstrating phase 2 reentry**. Accentuation of the phase 1 notch of the AP is seen in a heterogenous manner across epicardial sites. This leads to an exaggerated phase 1 notch and phase 2 dome at point **(A)**, whereas the phase 1 notch is accentuated to such an extent that the area is repolarised at point **(C)**, resulting in the loss of the phase 2 dome. This area is no longer refractory and therefore allows the phase 2 dome at point **(B)** to be conducted to epicardial site **(C)**. This results in extra systolic activity that manifests on the ECG as an R-on-T premature ventricular complex (PVC). This closely-coupled PVC initiates circus re-entry and PMVT/VF. The dispersion of the phase 1 notch manifests as J point elevation on the surface ECG.

Antzelevitch and colleagues have also provided valuable insights into the potential mechanisms of ventricular arrhythmia in ERS. Using the aforementioned canine model, with ventricular wedges from the inferior and lateral wall, they pharmacologically modeled ERS using a combination of I_to_ and I_K-*ATP*_ agonists and I_Ca_ and I_Na_ antagonists. These ionic currents were targeted based on evidence from the above studies as well genetic studies in ERS (discussed in subsequent sections). The authors demonstrated marked epicardial dispersion of repolarization in the ERS model, with a loss of the phase 2 AP dome and AP shortening in some epicardial regions but not others. Heterogeneous repolarization allowed propagation of the AP dome, resulting in local re-excitation, which manifested as closely coupled extrasystolic activity (phase 2 re-entry). The interaction between triggering extrasystoles and a ventricular substrate with marked repolarization heterogeneity resulted in VF (Koncz et al., 2014). See Figure 3 for a schema demonstrating phase 2 re-entry. The study also investigated the effects of vagal modulation and pharmacological therapy on arrhythmia susceptibility and described differences in arrhythmia susceptibility in the inferior and lateral left ventricle (discussed in detail in subsequent sections).

## Parallels with Brugada syndrome and the depolarization theory

Based on the above studies in animal models, ERS has widely been regarded as a repolarization abnormality, with J point elevation attributed to regional differences in the phase 1 AP notch. It is important to emphasize however that there remains controversy regarding the mechanistic basis of ERS. A competing theory proposes that ERS is a manifestation of a depolarization abnormality. The depolarization theory for ERS is largely based on parallels drawn with Brugada syndrome (BrS). BrS is characterized by J point elevation in the right precordial leads. Based on the clinical overlap between BrS and ERS, it has been proposed that BrS is a right ventricular variant of ERS (Benito et al., 2008; Haïssaguerre et al., 2008; Belhassen et al., 2009; Haïssaguerre et al., 2009b).

The mechanistic basis of BrS is also a subject of debate and the question regarding whether it is a repolarization abnormality or a depolarization abnormality remains unresolved (Meregalli et al., 2005). Indeed, uncertainty remains as to whether BrS is a primary channelopathy, a structural cardiac disease, or a combination of the two (Hoogendijk et al., 2010). The argument supporting BrS as a channelopathy is based on the identification of sodium channel mutations in BrS patients. However, it has been noted that such mutations are only present in ~20% of patients with BrS and the cause and effect of such mutations is not clear (Crotti et al., 2012). Some authors have therefore suggested that classifying BrS as a channelopathy is premature (Coronel et al., 2006). The argument supporting a structural cardiac disease is based on the identification right ventricular fibrosis and fatty infiltration, and wall motion abnormalities in BrS patients (Takagi et al., 2001; Frustaci et al., 2005; Zumhagen et al., 2009).

The depolarization theory for BrS proposes that a delay in activation in the right ventricular outflow tract (RVOT) results in a “current-to-load” mismatch, which manifests as J point and ST segment elevation (Hoogendijk et al., 2013). Evidence to support this theory is predominantly based on identification of fractionated electrograms and conduction slowing in the RVOT of BrS patients (Postema et al., 2008; Lambiase et al., 2009; Sacher et al., 2014). Studies in animal models have also demonstrated that sodium channel blockade results in excitation failure, manifesting as a BrS-type ST segment elevation (Hoogendijk et al., 2011). Based on computer modeling, it has been proposed that the *I*_to_ and *I*_CaL_ currents play important roles in modulating excitation failure.

There is a relative paucity of direct evidence to support the depolarization theory in ERS. In contrast to BrS patients, structural abnormalities (fibrosis and fatty infiltration), and conduction slowing (fractionated electrograms) have not been reported ERS patients. Based on responses to drugs such as quinidine and isoproterenol and genetic studies (discussed in the next section), the I_to_, I_CaL_, and I_Na_ currents have been implicated in ERS. Sequential activation of the I_Na_, I_to_, and I_CaL_ play important roles in depolarization and propagation of the AP(Nerbonne and Kass, 2005). Perturbations in these currents could therefore result in regional conduction delay and/or excitation failure in the inferior and lateral walls, resulting in an ER pattern. It is important to note however, that in contrast to BrS patients, ERS patients do not display significant ST segment elevation, which would argue against excitation failure in ERS. There is some evidence to suggest that conduction slowing without excitation failure may still lead to J-point elevation (Coronel et al., 2005). However, conduction slowing has not been demonstrated in patients with ERS thus far.

## Genetic studies and ionic currents implicated in ERS

**Figure 4:**
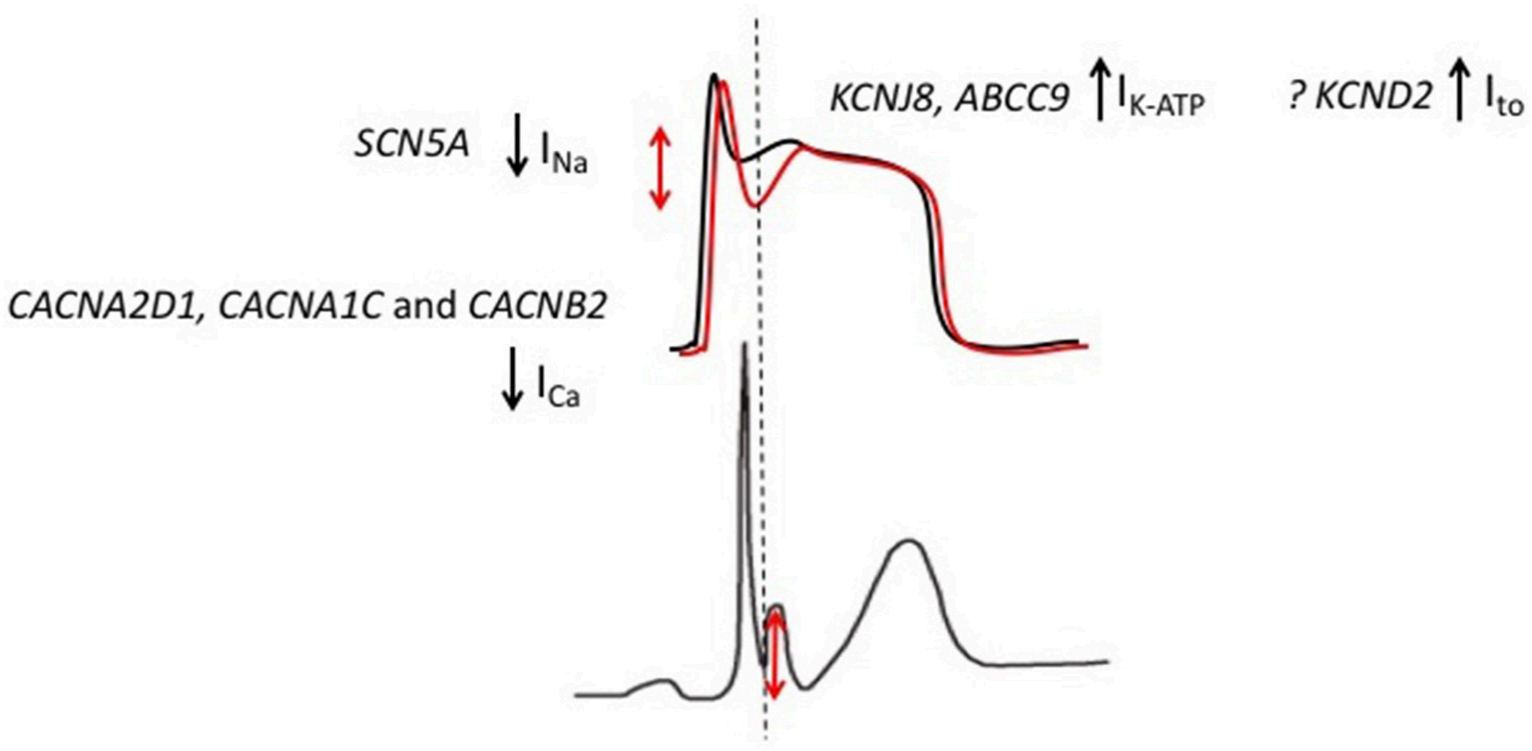
**Genetic mutations implicated in patients with ERS**. Gain-of-function mutations in KCNJ8 (ERS) and KCND2 (in an atypical J wave syndrome) result in augmented K_ATP_ and I_to_ currents, respectively. Loss-of-function mutations in *CACNA2D1, CACNA1C*, and *CACNB2* result in an attenuated I_Ca_ while SCN5A mutations are predicted to result in an attenuation of I_Na._The overall effect is an outward shift in the balance of currents, which is predicted to preferentially accentuate the epicardial AP notch (red).

Multiple ion channel mutations have been associated with ERS (Figure 4). The most frequently reported association has been between the KCNJ8 gene and ERS. *KCNJ8* encodes the K_ir_6.1 subunit of the K_ATP_ channel. Haissaguerre et al. reported a *KCNJ8* mutation in a case of ERS and VF storm (Haïssaguerre et al., 2009a). The same *KCNJ8* mutation (S422L) was observed by Medeiros-Domingo et al. in 2 out of a series of 101 patients with ERS and BrS. Both the affected cases had ERS. Functional characterization of the mutation revealed a gain-of-function in the K_ATP_ channel (Medeiros-Domingo et al., 2010). Barajas-Martinez et al. also identified the *KCNJ8* S422L mutation in 4 out of 204 ERS and BrS patients. They reported that the gain-of-function effect in K_ATP_ seen with this mutation is a consequence of a reduced sensitivity of the channel to intracellular ATP (Barajas-Martínez et al., 2012). More recently, mutations in *ABCC9*, which encodes the ATP-binding cassette transporter of I_K−ATP_ have been identified in ERS patients. After screening a cohort of 150 patients with ERS and BrS, Hu et al identified four ERS patients with *ABCC9* mutations (Hu et al., 2014). Functional analysis revealed that the mutation indirectly augments the I_K−ATP_ current.

As discussed in the previous section, the I_to_ current has been implicated as an important contributor to J wave syndromes in animal studies. On the basis of these reports, Perrin et al. performed genetic screening in a cohort of 31 ERS patients and 20 Brugada syndrome patients for mutations in genes encoding I_to_ channel subunits (*KCNA4, KCND2*, and *KCND3*). They also screened the KCNJ8 gene. They did not identify mutations in any patients with infero-lateral ERS. A single mutation in *KCND2* (D612N) was identified in a patent with an unusual J wave syndrome with evidence of QRS notching in the anterior leads. Functional characterization revealed a gain-of-function effect with an increased I_to_ current density.

Mutations in ion channels that encode inward currents have also been implicated in ERS. Burashnikov and colleagues reported multiple mutations in L-type calcium channel subunit genes (*CACNA2D1, CACNA1C*, and *CACNB2*) after screening 24 ERS patients (Burashnikov et al., 2010). Preliminary functional analysis revealed that the mutations are associated with loss-of-function type modulation (Napolitano and Antzelevitch, 2011). Watanabe et al. identified mutations in the SCN5A gene in three ER patients after screening a cohort of 26 patients. Functional studies demonstrated a loss-of-function effect, with all three mutations failing to generate an I_Na_ current (Watanabe et al., 2011).

The findings from genetic studies are generally consistent with the repolarization theory for ERS. Although, it should be noted that only one mutation has been identified in the I_to_ channel. The I_to_ and I_K−ATP_ currents play important roles during the early phases of the AP and are more abundantly expressed in the epicardium relative to the endocardium. Therefore, gain-of-function mutations in I_to_ and I_K−ATP_ are predicted to increase the transmural voltage gradient by preferentially augmenting the epicardial phase 1 notch (Antzelevitch and Barajas-Martinez, 2010; Benito et al., 2010). Loss-of-function I_Ca_ and I_Na_ channel mutations on the other hand are predicted to reduce depolarizing forces. As a result of higher epicardial expression of the aforementioned I_to_ and I_K−ATP_ currents, the epicardium is more susceptible to the reduction in depolarizing forces (Meregalli et al., 2005). Therefore, loss-of-function I_Ca_ and I_Na_ channel mutations are also predicted to augment transmural voltage gradients during the early phases of the AP. Overall, gain-of-function I_K−ATP_ and I_to_ channel mutations and loss-of-function I_Ca_ and I_Na_ channel mutations are predicted to result in an overall outward shift in the balance of currents, which in turn is predicted to accentuate the epicardial AP notch Figure 4; (Koncz et al., 2014; Patocskai et al., 2016). Of note, based on results from genetic studies, pharmacological augmentation of I_K−ATP_ and I_to_ and attenuation of I_Ca_ and I_Na_ have been used to recapitulate both the ECG pattern and arrhythmic features of ERS in a canine ventricular wedge model (Koncz et al., 2014).

The reported genetic mutations may also support the depolarization theory for ERS. Loss-of-function I_Na_ channel mutations are predicted to directly reduce conduction velocity by decreasing the AP upslope. I_to_ and I_Ca_ channel mutations also have the potential to influence conduction velocity by altering the early phase of the AP (Yan and Antzelevitch, 1999; Hoogendijk et al., 2011, 2013). Evidence to support this theory has come from a recent study from Hoogendijk and colleagues. In porcine *ex vivo* model, they demonstrated that sodium channel blockade results in excitation failure and ST elevation on a pseudo ECG. Furthermore, using computer modeling, they demonstrated that manipulation of the I_to_ and I_Ca_ channels also influences current-to-load matching (Hoogendijk et al., 2011). Specifically, *I*_to_ reduced the current available for conduction while, *I*_CaL_ had the opposite effect. On the basis of these observations, it is plausible that the reported mutations influence the ERS phenotype through altered depolarization.

## Clinical correlations

As discussed in previous sections, the repolarization theory regarding the mechanistic basis of ERS proposes that J point elevation is a manifestation of augmented heterogeneity of the AP notch, which predisposes to phase 2 re-entry and ventricular arrhythmia. The depolarization theory on the other hand proposes that J point elevation is a manifestation of delayed activation of specific regions of the myocardium, a “current-to-load” mismatch. To date, the evidence in support of the repolarization theory has been more compelling.

**Figure 5:**
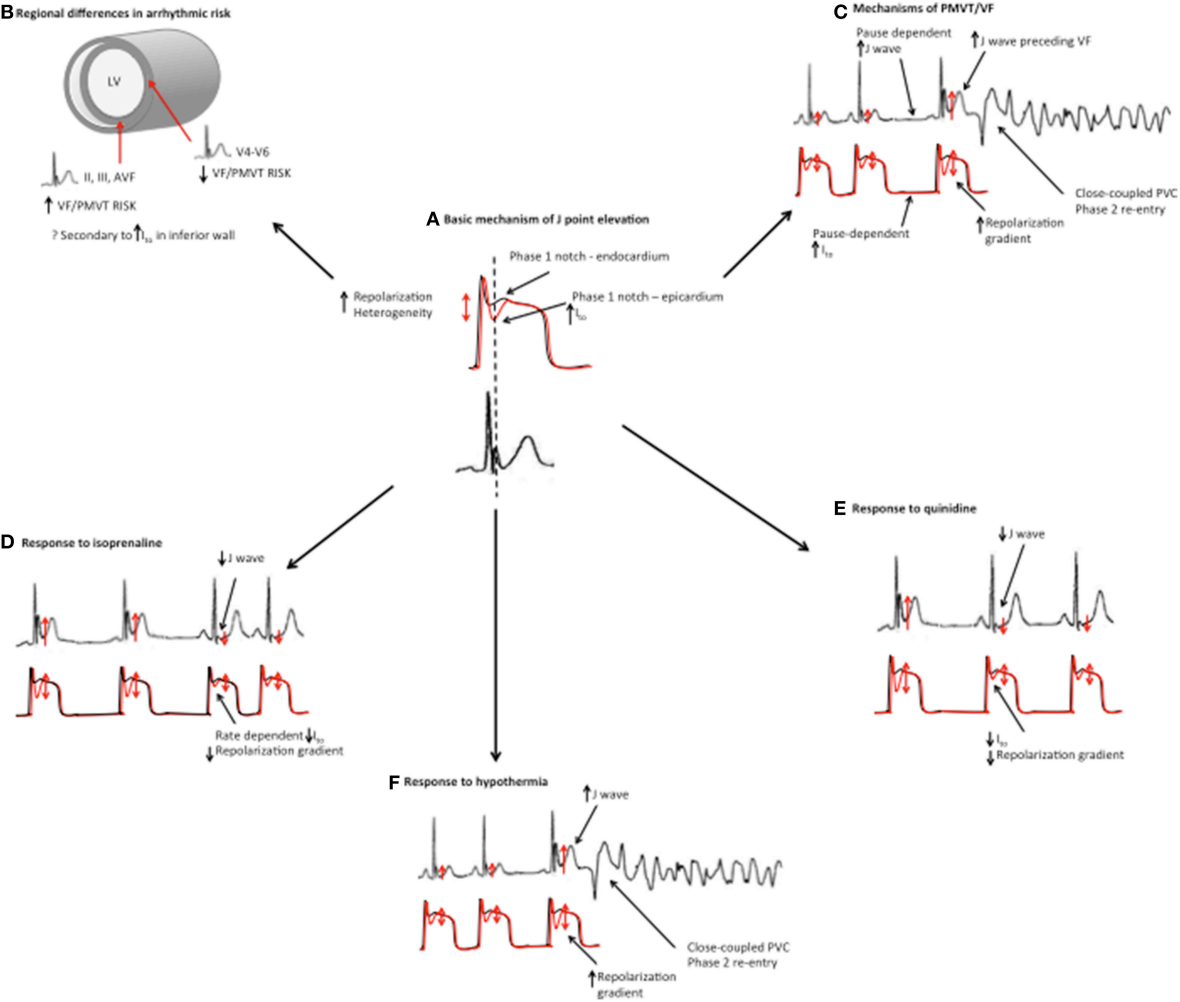
**Mechanistic links between basic science studies and clinical features in ERS**. **(A)** Basic science studies have demonstrated that J point elevation is a manifestation of increased transmural repolarization heterogeneity. **(B)** Patients with an inferior ER pattern have a higher risk of ventricular arrhythmias, which could potentially be related to increased I_to_ in the inferior wall. **(C)** ERS patients demonstrate more pronounced J point elevation immediately preceding VF episodes. Higher J waves may reflect augmented repolarization heterogeneity. J wave augmentation is pause-dependent, which may, be a reflection of I_to_ channel kinetics (slow temporal recovery from activation). The proposed mechanism of VF/PMVT is phase 2 reentry. **(D)** Isoproterenol suppresses VF/PMVT and reduces J point elevation in ERS, an effect that is thought to be mediated by elevated heart rates and reduced I_to_ channel recovery. **(E)** Quinidine also suppresses VF/PMVT in ERS by blocking the I_to_ current. **(F)** Hypothermia results in an augmented transmural repolarization gradient and an increased susceptibility to VF/PMVT.

Since the reports of an association between ER and SCD, multiple clinical studies have described the characteristics of ER patients who have had VF. The majority of clinical observations have been correlated with the repolarization theory (Figure 5). There are however some aspects of the clinical syndrome that may support the depolarization theory. There also remain some clinical features of ERS that are unexplained. The correlation between mechanistic theories and the clinical syndrome is discussed below.

### Dispersion of repolarization and phase 2 re-entry

In ERS patients with electrical storm, Nam et al. reported a marked increase in the J wave amplitude prior to the onset of VF. They also identified closely coupled ventricular premature beats (VPBs) triggering VF episodes. The authors recorded the T_peak_-T_end_, a marker of repolarization in these patients and found significant differences in the T_peak_-T_end_ between ECG leads around the time of VF (Nam et al., 2010). These clinical observations are consistent with increased dispersion of repolarization and phase 2 re-entry preceding VF episodes, which correlates with an accentuation of the phase 1 AP notch preceding phase 2 re-entry in animal models (Patocskai et al., 2016).

Aizawa and colleagues also reported that patients with ERS display higher J wave amplitudes preceding VF episodes. Moreover, they reported pause-dependent accentuation of J point elevation preceding VF episodes (Aizawa et al., 2012). These observations are consistent with cellular electrophysiology studies demonstrating slow temporal recovery of the I_*to*_ channel from inactivation, which results in an augmented *I*_to_ current at slower heart rates (Yan and Antzelevitch, 1996). In keeping with the *I*_to_ channel kinetics, administration of acetylcholine has been reported to increase the phase 1 AP notch which in turn increases dispersion of repolarization and susceptibility to VF (Koncz et al., 2014). These data may also explain why VF is more frequently observed in patients with ERS during sleep or low levels of activity (Antzelevitch and Yan, 2015).

Invasive mapping studies in patients with ERS using monophasic action potential (MAP) catheters have demonstrated more prominent phase 1 AP notching in the inferior and lateral left ventricular epicardium (Mahida et al., 2015). These findings provide direct support for basic science reports demonstrating that a prominent epicardial phase 1 AP notch underlies J point elevation (Koncz et al., 2014). Invasive studies have also reported that amongst patients with an inferior ER pattern, short-coupled VPBs that potentially trigger VF originate from the inferior wall of the left ventricle (Haïssaguerre et al., 2008). These observations are consistent with studies in *ex vivo* models of ERS demonstrating that local re-excitation (phase 2 re-entry) and closely coupled extrasystolic activity, occurs in regions with augmented dispersion of repolarization.

### Arrhythmic risk in inferior vs. lateral ER

The distribution of J point elevation has been proposed to influence risk of VF in ERS. Multiple clinical studies have demonstrated that J point abnormalities in the lateral ECG leads are associated with the lowest risk of VF whilst J point abnormalities in the inferior leads and global J point abnormalities are associated with progressively increasing risk of VF (Antzelevitch and Yan, 2010). These findings are consistent with animal studies demonstrating higher levels of I_to_ in the inferior wall of canine myocardium and in experimental conditions that mimic ERS, this region of myocardium is more vulnerable to VT/VF (Koncz et al., 2014). Further evidence has come from studies demonstrating a higher expression of I_to_ channel subunits in the inferior wall of the canine LV (Szentadrassy et al., 2005). It should be noted, however, that although current data offers explanation as to why an inferior pattern confers a higher risk of VF, they do not explain why an ER pattern in the lateral leads does not increase risk despite J point elevation.

### Arrhythmic risk in horizontal vs. downsloping ST segments

A horizontal or downsloping morphology of the ST segment has been reported to be a marker of increased arrhythmic risk in cohorts of patients with the ER pattern (Tikkanen et al., 2011). To our knowledge there is no mechanistic data to explain this phenomenon. There are, however, some parallels that can be drawn from the BrS. Provocative testing in BrS patients with class 1c drugs can result in a type 1 ECG, with downsloping ST segment elevation being characteristic. It could be speculated that the horizontal/downsloping ST segments observed in ERS are analogous to those seen in BrS and are therefore representative of similar cellular and ionic abnormalities. It is important to note however that this proposed link is speculative; while downsloping ST segments are occasionally observed in ERS the T wave inversion observed in BrS are not seen in ERS.

### Late potentials

A relatively high incidence if late potentials (LP) on signal-averaged ECG (SAECG) has been reported in ERS patients (Abe et al., 2010). The LP have been reported to demonstrate dynamic circadian changes, with more pronounced LP parameters at night time. LP are thought to represent areas of delayed ventricular conduction. Therefore, the identification of late potentials in ERS may support the depolarization theory of ERS. Further evidence to support this notion comes from studies in BrS patients. Invasive studies in BrS patients have demonstrated that LP on the SAECG are correlated with delayed local electrograms recorded during electrophysiology studies (Nagase et al., 2002). As discussed previously however, extrapolating results from BrS to ERS should be performed with caution.

### Pharmacological responses

Quinidine has been demonstrated to suppress VF storm in patients with ERS (Haïssaguerre et al., 2009a,b). One of the first reports on the efficacy of quinidine in ERS came from Haïssaguerre et al. (2009a). In a young patient with VF storm that was refractory to multiple antiarrhythmic drugs, only quinidine was effective at preventing VF. In a subsequent multicentre study, the same group demonstrated that quinidine effectively suppressed arrhythmia in 9 out of 9 ERS patients with VF storm (Haïssaguerre et al., 2009b). Their clinical observations are in keeping with basic science studies. Quinidine has consistently been demonstrated to abrogate the ER pattern, and the associated exaggerated phase 1 notch in *in vitro* models of ERS (Koncz et al., 2014; Szél and Antzelevitch, 2014). Quinidine is predicted to exert its antiarrhythmic effect in ERS patients by suppressing the I_to_ current (Antzelevitch and Yan, 2015).

Isoproterenol has been shown to effectively suppresses electrical storm in ERS patients in multiple studies (Haïssaguerre et al., 2009a). Furthermore, isoproterenol consistently normalizes J waves in patients with lateral ERS. In patients with inferior ERS, the response to beta adrenergic stimulation was more heterogenous (Roten et al., 2012a). Consistent with these clinical observations, studies in canine models have demonstrated that isoproterenol normalizes J point elevation (Yan and Antzelevitch, 1999). The effect of isoproterenol is likely to be mediated by a combination of heart rate acceleration, which is predicted to reduce *I*_to_, and an increase in *I*_Ca_, that restores the phase 2 dome and hence transmural AP homogeneity (Koncz et al., 2014; Patocskai et al., 2016). The distinctive and heterogenous clinical response of inferior ER to isoproterenol remains unclear. As discussed previously, I_to_ expression has been reported to be higher in the inferior wall (Koncz et al., 2014). Therefore, isoproterenol would be expected to have a homogenous effect in inferior ERS patients. A potential explanation may relate to the fact that the study included a heterogenous cohort of patients which included patients with VF, patients with syncope and asymptomatic patients. Therefore, not all patients in the study had the malignant form of ERS.

In addition to the reported efficacy of quinidine and isoproterenol in the aforementioned studies, a number of antiarrhythmic drugs including beta-blockers, lidocaine, mexiletine, and verapamil were demonstrated to be ineffective (Haïssaguerre et al., 2009b). Supporting evidence for these observations has come from both basic science and genetic studies. Beta-blockers and verapamil slow the heart rate, which is predicted to increase I_to_ channel recovery from inactivation (Yan and Antzelevitch, 1996). In turn, enhanced I_to_ channel recovery increases the phase 1 AP notch and dispersion of repolarization. In the specific case of verapamil, based on studies in canine models, calcium channel blockade leads to a loss of the phase 2 dome of the AP, an effect that is also predicted to enhance dispersion of repolarization (Fish and Antzelevitch, 2004). The failure of sodium channel blockers such as lidocaine and mexiletine to suppress arrhythmia in ERS is consistent with the reports of loss-of-function *SCN5A* mutations underlying ERS and animal studies demonstrating that *I*_Na_ blockade with pilsicainide increases susceptibility to VF (Watanabe et al., 2011; Koncz et al., 2014). Overall, the observations from basic science studies predict that beta-blockers, sodium channel blockers, and calcium channel blockers could actually have a proarrhythmic effect in ERS.

As discussed previously, ERS demonstrates significant overlap with BrS, which is typically associated with an accentuated J wave following administration of the sodium channel blocker ajmaline (Antzelevitch and Yan, 2015). Based on genetic studies reporting loss-of-function *SCN5A* mutations in ERS and basic science studies demonstrating more prominent AP notching and J waves following *I*_Na_ blockade, ajmaline would be predicted to accentuate J waves in ERS patients. Paradoxically however, a number of studies have reported that ajmaline attenuates J waves (Kawata et al., 2012; Roten et al., 2012b). Of note, this response to ajmaline has not been consistent in all studies. A recent case report involving epicardial left ventricular mapping in an ERS patient demonstrated that administration of pilsicainide, an *I*_*Na*_ blocker, resulted in augmentation of epicardial J waves in the lateral left ventricle. This change was however not observed on the surface ECG (Nakagawa et al., 2014). The mechanisms underlying these discrepant observations remain to be elucidated.

### Hypothermia and VF in ERS

The association between hypothermia and J point elevation has been recognized for decades. In 1938, Tomaszewski et al. described a case of a frozen man with J point elevation (Tomaszewski, 1938). Interestingly, hypothermia has been reported to increase the risk of ventricular arrhythmias in patients with ERS. Bastiaenen et al. reported a case of inferior ER who developed VF storm with more pronounced J point elevation in response to therapeutic hypothermia (Bastiaenen et al., 2010). Federman and colleagues reported a case of infero-lateral ER with hypothermia-induced VF storm (Federman et al., 2013). The association between hypothermia and J point elevation is also well established in animal models (Osborn, 1953; Santos and Kittle, 1958; Emslie-smith et al., 1959; West et al., 1959). In a canine model of ERS, hypothermia has been reported to accentuate the epicardial AP notch, which results in a heterogeneous loss of the AP dome, phase 2 re-entry and VF (Gurabi et al., 2014). Based on the clinical and basic science findings, it could be speculated that hypothermia acts as an additional “insult” in ER patients with an underlying arrhythmogenic substrate.

### ER as a risk predictor in coronary artery disease

The ER pattern has been associated with an increased risk of arrhythmic events in patients with coronary artery disease. Multiple previous studies have demonstrated that in patients with acute myocardial infarction, the presence of ER is associated with a significantly increased of ventricular arrhythmia (Naruse et al., 2012; Rudic et al., 2012; Tikkanen et al., 2012) ER has also been associated with an increased risk of ventricular arrhythmia in patients with chronic coronary artery disease (Patel et al., 2010).

Interestingly, the mechanisms of ventricular arrhythmia in patients with acute myocardial infarction are thought to be similar to those in patients with ERS. Yan and colleagues have previously demonstrated that in an *ex vivo* right ventricular wedge preparation, regions of ischaemia had a loss of the *I*_to_-mediated epicardial AP dome with phase 2 re-entry resulting in VF (Di Diego and Antzelevitch, 2011; Yan et al., 2004). As discussed previously, a similar mechanism has been reported by the same group in an *ex vivo* model of ERS. On the basis of these observations, it could be speculated that ER augments arrhythmic risk in acute myocardial infarction by increasing risk of phase 2 re-entry (Naruse et al., 2012).

It is important to note that in patients with coronary artery disease, particularly chronic coronary artery disease, the presence of J point elevation in the inferior could potentially be a manifestation of delayed depolarization due to “peri-infarction block” in areas of scar (Castle, 1965; Littmann, 2010; Tikkanen et al., 2012). Indeed, as discussed previously, the ER pattern could also potentially be a manifestation of a depolarization abnormality. Overall, there remains significant uncertainty regarding the mechanistic links between ER and increased risk of ventricular arrhythmia in patients with coronary artery disease.

### False tendons and ER

The ER pattern is more prevalent in patients with false tendons (Nakagawa et al., 2012). False tendons are fibromuscular bands containing conduction tissue which traverse the LV cavity (Luetmer et al., 1986; Abdulla et al., 1990; Loukas et al., 2007). The mechanistic link between false tendons has yet to be elucidated. It has been proposed that false tendons may create localized stretching, which in turn results in repolarization gradients and J point elevation. An alternative hypothesis is that false tendons result in altered conduction to the inferior and lateral walls, leading to J point elevation (Nakagawa et al., 2012). It is plausible therefore that J point elevation in patients with false tendons is a manifestation of an intraventricular conduction defect or repolarization abnormality. However, at this stage, these proposed mechanisms remain speculative.

## Future directions

Despite significant advances in basic science studies, important gaps remain in our understanding of the mechanistic basis of ERS. Current mechanistic evidence is based primarily on studies in an *ex vivo* model of ERS, and debate continues with regards the fundamental mechanistic abnormality that underpins ERS, i.e., repolarization or depolarization. While these *ex vivo* studies have provided valuable insights, a degree of caution must be exercised when extrapolating results to a clinical situation. In the future, more refined basic science and translational studies are necessary to further our understanding of the mechanistic basis of ERS. Potential areas of future research are outlined below.

To date, classical genetic studies in ERS have identified a small number of ion channel mutations. The mechanistic link between these mutations and ERS has not been fully characterized. The recent emergence of next-generation sequencing technologies such as exome sequencing and whole-genome sequencing has significantly enhanced our ability to identify the genetic basis of inherited arrhythmia syndromes. The application of these technologies in ERS patients is predicted to identify additional causative mutations. In terms of functional characterization of the genetic variants, induced pluripotent stem cell (iPS) technology represents a promising novel technique. Patient-specific iPS cells have successfully recapitulated the cellular electrophysiology of a number of inherited arrhythmia syndromes (Moretti et al., 2010; Itzhaki et al., 2011; Yazawa et al., 2011). The application of iPS technology in genotype-positive ERS patients is likely to enhance our understanding of the mechanistic basis of the trait.

Electrocardiographic imaging ECGI is an emerging translational research tool for inherited arrhythmia syndromes (Zhang et al., 2015). The technique involves recording body surface potentials using 250 body surface electrodes and computing potentials on the epicardium using the inverse solution (Rudy and Lindsay, 2015). Information from epicardial potentials is subsequently used to derive information on ventricular activation and repolarization. Ghosh et al. recently utilized ECGI in ERS patients to demonstrate early repolarization in regions of the LV that corresponded to the ECG leads with an ER pattern (Ghosh et al., 2010). These regions also had steep activation recovery interval (ARI) gradients, which are predicted to promote re-entrant arrhythmias (Ghosh et al., 2010). In the future, studies in larger numbers of ERS patients with different ER patterns, e.g., horizontal and downsloping ST segments, may provide valuable mechanistic information. It is important to note that while ECGI has demonstrated early promise, it is associated with significant limitations. For a given set of body surface potentials, there exist multiple potential cardiac solutions when the inverse solution is applied. Therefore, assumptions have to be applied to the analysis. Furthermore, small errors in the recorded body surface potentials can result in large errors in the computed epicardial potentials (Rudy and Lindsay, 2015).

To date, the majority of mechanistic studies in ERS have focused on ion channels and their potential contribution to abnormal repolarization and/or depolarization. In addition to ion channels, proteins that mediate cell-to-cell coupling, such as connections may play important roles in the pathogenesis of ERS. Interestingly, Nademanee and colleagues demonstrated that patients with BrS have reduced RVOT expression of connection-43, which is a major determinant of electrical coupling and conduction velocity in the ventricle (Nademanee et al., 2015). In the future, research into connnexin function in patients with ERS may provide important insights into the mechanistic basis of the trait.

## Conclusions

Our understanding of the ERS has evolved rapidly in recent years but significant questions and controversies remain. The body of basic scientific data from animal and human studies continues to grow and there is evidence that developing electrophysiological and imaging technologies are beginning to bridge the gap between the bench and the bedside. These are important developments in terms of enhancing risk stratification as well as developing effective treatment strategies.

## Author contributions

BM, manuscript writing; GAB, critical revisions; SP, critical revisions; CB, critical revisions; MT, critical revisions; SM, manuscript writing and critical revisions

### Conflict of interest statement

The authors declare that the research was conducted in the absence of any commercial or financial relationships that could be construed as a potential conflict of interest.
